# Liver metastases - An unusual cause of portal hypertension: A case report

**DOI:** 10.1016/j.amsu.2022.104912

**Published:** 2022-11-16

**Authors:** Rajesh Pandey, Sambhu Khanal, Shashank Neupane, Bishal Dhakal, Prasamsa Pudasaini, Sabina Khadka, Rupika Adhikari, Bipin Poudel

**Affiliations:** a Department of Internal Medicine, Civil Service Hospital, Kathmandu, Nepal; b Department of Internal Medicine, Lumbini Provincial Hospital, Butwal, Nepal; c Nepalese Army Institute of Health Sciences, Sanobharyang, Kathmandu, Nepal; d Bharatpur Hospital, Chitwan, Nepal

**Keywords:** Liver metastases, Breast carcinoma, Complication, Portal hypertension

## Abstract

**Introduction:**

Portal hypertension is a rare complication of liver metastases. The study highlights that clinician should be aware of conditions mimicking cirrhosis with similar clinical presentation and imaging findings.

**Case presentation:**

We present the case of a 29-year-old non-alcoholic lady who presented to our hospital with a history of two months of progressive, painless abdominal distension and progressively increasing yellowish discoloration of the eyes. Physical examination, laboratory investigations, and imaging tests led to a diagnosis of multiple metastases from breast carcinoma to the liver leading to portal hypertension after exclusion of other causes of portal hypertension. However, after three weeks of presentation to the hospital, the patient died before any therapeutic measures were initiated to address breast carcinoma.

**Clinical discussion:**

Liver metastasis from primary breast carcinoma rarely presents with clinical symptoms of portal hypertension. Although portal hypertension secondary to pseudocirrhosis, predominantly linked to ongoing chemotherapy for known cancers, has been previously described in case studies, our case had an unusual presentation leading to diagnostic uncertainty.

**Conclusion:**

Our case highlights the rare cause of liver metastasis secondary to breast carcinoma, which presented as portal hypertension.

## Highlights

• Portal hypertension is a rare complication of liver metastases, frequently occurring in malignant diseases.
• In patients with liver metastases, chemotherapy can result in areas of retracted tumor tissue and scarring, also known as pseudocirrhosis.
• Although portal hypertension is secondary to pseudocirrhosis and predominantly linked to ongoing chemotherapy for known cancers, our case had an unusual presentation leading to diagnostic uncertainty.
• The patient has an atypical presentation with symptoms consistent with portal hypertension and chronic liver disease without prior exposure to chemotherapy which could be due to the tumor deposits resulting in sinusoidal obstruction, subsequently leading to portal hypertension.

## 1 Introduction

Portal hypertension is a rare complication of liver metastases, frequently occurring in malignant diseases [1]. Although portal hypertension secondary to pseudocirrhosis, predominantly linked to ongoing chemotherapy for known cancers, has been previously described in case studies [[2], [3], [4], [5]], our case had an unusual presentation leading to diagnostic uncertainty. Unlike portal hypertension in liver cirrhosis, portal hypertension in liver metastasis has been attributed to the increase in portal flow resistance at any site within the portal venous system due to mechanical obstruction [1]. The study highlights that clinician should be aware of conditions mimicking cirrhosis. Although rare, liver metastases can be an unusual cause of portal hypertension. The case has been reported as per SCARE 2020 guidelines [12].

## 2 Case presentation

Here, we present a 29-year-old lady who presented to our hospital with a history of two months of progressive, painless abdominal distension and progressively increasing yellowish discoloration of the eyes. She also had generalized weakness and weight loss of 4.5 kg in the same duration. She denied changes to appetite or bowel habits, melena, hematemesis, and altered sensorium. She had no personal or family history of chronic liver disease, no history of alcohol, or use of any drug or alternative medicine. Besides jaundice, there were no peripheral stigmata of chronic liver disease. Abdominal examination revealed a grossly distended abdomen with fluid thrill consistent with ascites. Similarly, examination of breast revealed a hard, non-tender irregular mobile mass measuring 3cm × 4 cm on the left upper quadrant of the left breast and another hard, non-tender, immobile irregular mass measuring 3cm × 2cm in the lower quadrant of the right breast.

Liver function tests were deranged with impaired synthetic function. Viral screening and autoantibody tests were negative. Serum Ceruloplasmin level was normal. She had normal alpha-fetoprotein but elevated cancer antigen 125 and carcinoembryonic antigen. Laboratory findings are listed in Table 1. Ascitic fluid analysis showed high SAAG (1.7g/dl) and low protein (1.2 g/dl) ascites. Three samples of ascitic fluid for malignant cytology were negative. UGI Endoscopy showed Grade 2 oesophageal varices without any red colour signs and mild portal hypertensive gastropathy.

**Table 1:** Laboratory findings.

Laboratory tests	Result	Unit	Reference range
Total Leukocytes Count (TLC)	8.4	10^˄^3/μL	4–11
Neutrophil	62	%	40–80
Lymphocyte	18	%	20–40
Hemoglobin	12.4	g/dl	13–17
Platelet Count	166	10^˄^3/μL	150–450
Urea	29.3	mg/dl	17–43
Creatinine	0.9	mg/dl	0.7–1.3
Sodium	137	mEq/L	135–145
Potassium	4.0	mEq/L	3.5–5.5
Bilirubin Total	136	micromole/l	1.71–20.5
Bilirubin Direct	100	micromole/l	<5.1
Alkaline Phosphatase (ALP)	620	U/L	53–128
Alanine Transferase (ALT)	185	U/L	0–35
Aspartate Transferase (AST)	778	U/L	0–35
Serum Albumin	29	g/dl	3.4–5.4
Prothrombin time (PT)/International Normalized Ratio	21/1.75	seconds	11–13.5
Hepatitis B Surface antigen	Negative		
Hepatitis C Antibody	Negative		
Human Immunodeficiency Virus	Negative		
Antinuclear Antibody	Negative		
Liver kidney microsomal type 1 antibody	Negative		
Anti-smooth muscle antibody	Negative		
Anti-mitochondrial antibody	Negative		
Ceruloplasmin	33.20	mg/dl	14–40
Transferrin Saturation	27.59	%	15–50
Alpha Fetoprotein	2.53	ng/ml	10–20
Cancer Antigen-125	685	U/ml	0–35
Carcinoembryonic Antigen	>400	ng/mL	0–2.5

**Fig. 1:**
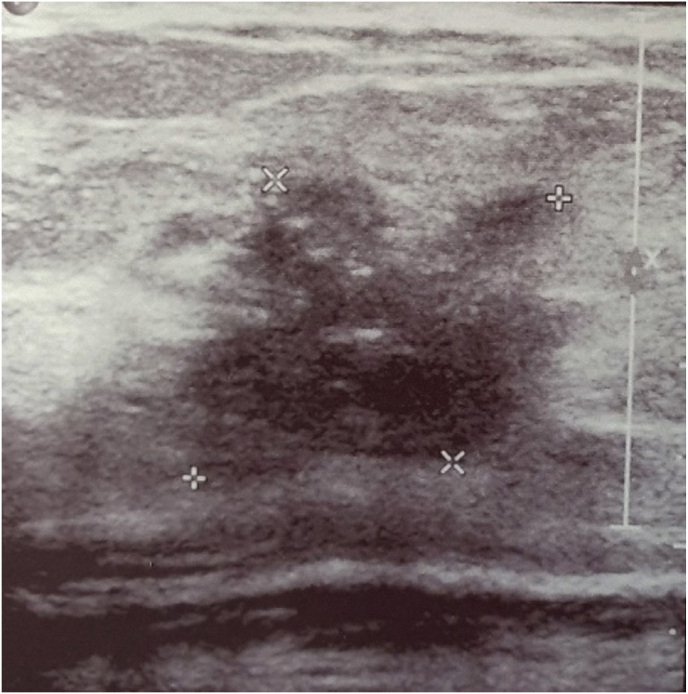
Ultrasound abdomen. Legend: Spiculated, hypoechoic lesion (2.5 × 2.2 × 1.8 cm) with calcification in the right breast.

Abdominal ultrasound showed hepatomegaly with coarse echotexture and a few well-defined hyperechoic nodules with surrounding hypoechoic rim, ascites, and a patent portal vein without splenomegaly. The chest X-ray was normal. Breast ultrasound showed spiculated, hypoechoic lesion (2.5 × 2.2 × 1.8 cm) (Fig. 1) in the right breast suspicious of malignancy and multiple well-defined hypoechoic oval lesions in bilateral breasts. Tru-cut biopsy from the right breast showed nests, cords, and a few tubules of tumor-infiltrating the fibro adipose tissue, which was suggestive of infiltrating duct carcinoma (Nottingham Histological score 8, Grade 3), while a tru-cut biopsy from the left breast was suggestive of a fibroepithelial tumor.

**Fig. 2:**
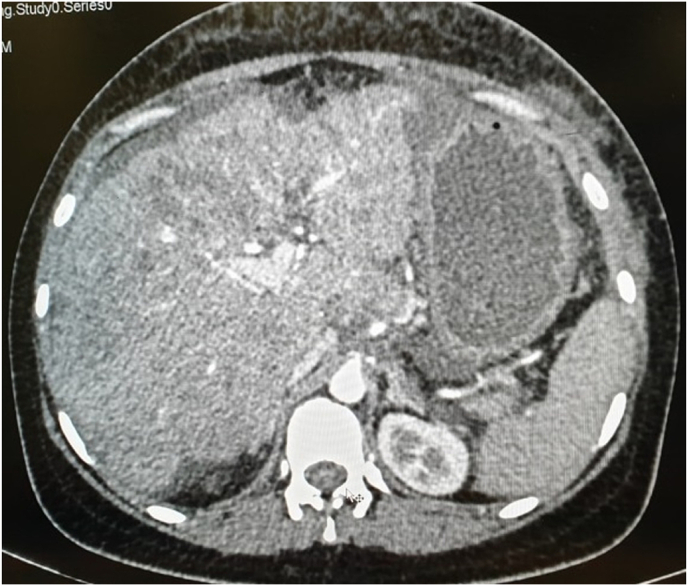
CT arterial phase (Liver). Legend: An irregular liver with several hypoechoic lesions with slight enhancement and ascites.

**Fig. 3:**
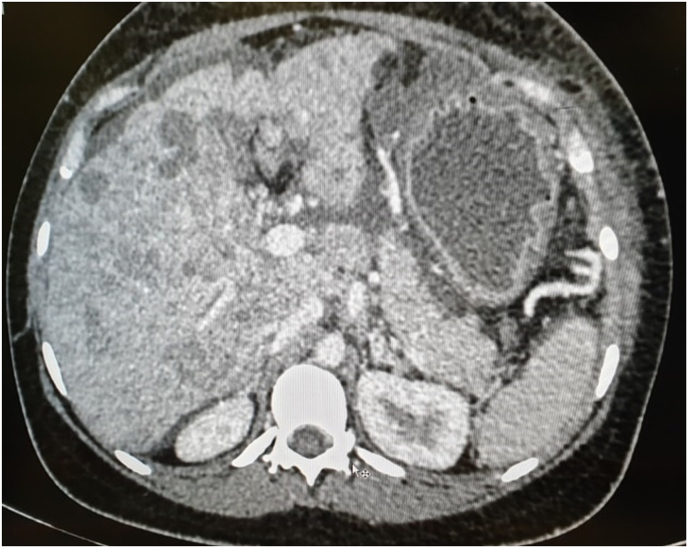
CT Venous phase. Legend: Ascites.

**Fig. 4:**
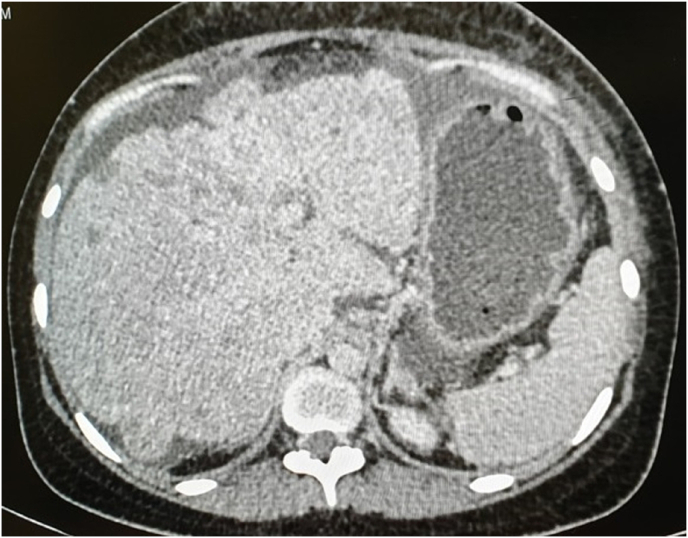
CT showing washout in delayed phase and ascites. Legend: Washout in delayed phase and ascites.

Triple phase CECT showed the enlarged and irregular outline of the liver with multiple variable-sized hypodense lesions with slight enhancement in the arterial phase and washout in the delayed phase with the dilated portal vein (13.4mm) and gross ascites but no splenomegaly (Fig. 2, Fig. 3, Fig. 4).

However, the patient died after three weeks of presentation to the hospital before any therapeutic measures were initiated concerning the liver metastasis, with the primary being breast carcinoma.

## 3 Discussion

Patients with breast cancer and liver metastasis rarely present with clinical symptoms of portal hypertension; abdominal distension with ascites is the most common presenting complaint [5]. It has also been reported in oesophageal [6], pancreatic [7], colorectal [8], and thyroid cancers [9].

In patients with liver metastases, chemotherapy can result in areas of retracted tumor tissue and scarring, also known as pseudocirrhosis (resembling cirrhosis radiographically but lacking pathologic features), which can also lead to hepatic decompensation [2]. Pseudocirrhosis is mainly associated with the progression of malignancy receiving chemotherapy [10]. Several agents are associated with pseudocirrhosis in patients with breast cancer, like adriamycin, cyclophosphamide, 5-fluorouracil, methotrexate, cisplatin, tamoxifen, paclitaxel, vinblastine, etoposide, and vincristine [4,11]. This case illustrates an atypical presentation with symptoms consistent with portal hypertension and chronic liver disease without prior exposure to chemotherapy. This could be due to the tumor deposits resulting in sinusoidal obstruction, subsequently leading to portal hypertension [10]. This is the first reported case of pseudocirrhosis due to liver metastasis from breast carcinoma in Nepal.

## 4 Conclusions

The case we have presented highlights the need for clinicians to be aware of conditions mimicking cirrhosis. Although rare, liver metastases can be an unusual cause of portal hypertension.

## Ethical approval

N/A.

## Sources of funding

None.

## Authors contributions

Author 1: Led data collection, the concept of the study, contributed to writing the case information.

Author 2: Literature review and writing case information.

Author 3: Literature review, revising, and editing the manuscript into the final version.

Author 4: Literature review, revising and editing the manuscript.

Author 5: Literature review, revising and editing the manuscript.

Author 6: Literature review, revising and editing the manuscript.

Author 7: Literature review, revising and editing the manuscript.

Author 8: Literature review, revising and editing the manuscript.

All authors were involved in manuscript drafting and revising, and approved the final version.

## Research registration

N/A.

## Guarantor

Mr. Shashank Neupane, Nepalese Army Institute of Health and Sciences, Sanobharyang, Kathmandu, Nepal. E-mail: shashankneupane5107@gmail.com.

## Consent

Written informed consent was obtained from the patient for publication of this case report and accompanying images. A copy of the written consent is available for review by the Editor-in-Chief of this journal on request.

## Provenance and peer review

Not commissioned, externally peer-reviewed.

## Declaration of competing interest

None.

## References

[1] Theophilidou E., Waraich N., Raza T., Agarwal P.K. (2012). Liver metastases, a rare cause of portal hypertension and stoma bleeding. Brief review of literature. Int J Surg Case Rep.

[2] Young S.T., Paulson E.K., Washington K. (1994). CT of the liver in patients with metastatic breast carcinoma treated by chemotherapy: findings simulating cirrhosis. AJR Am. J. Roentgenol..

[3] Kashyap R., Reddy R., Voona M.K. (2018). Pseudocirrhosis of the liver in setting of metastatic carcinoma breast: an ominous sign to be remembered. Indian J. Nucl. Med..

[4] Qayyum A., Lee G.K., Yeh B.M. (2007). Frequency of hepatic contour abnormalities and signs of portal hypertension at CT in patients receiving chemotherapy for breast cancer metastatic to the liver. Clin. Imag..

[5] Adike A., Karlin N., Menias C., Carey E.J. (2016). Pseudocirrhosis: a case series and literature review. Case Rep Gastroenterol.

[6] Kobashigawa C., Nakamoto M., Hokama A. (2010). Pseudocirrhosis in metastatic esophageal cancer. South. Med. J..

[7] Kang S.P., Taddei T., McLennan B., Lacy J. (2008). Pseudocirrhosis in a pancreatic cancer patient with liver metastases: a case report of complete resolution of pseudocirrhosis with an early recognition and management. World J. Gastroenterol..

[8] Battisti S., Guida F.M., Pagliara E. (2014). Pseudocirrhosis after anti-EGFR-based neoadjuvant therapy for hepatic metastasis from colon cancer: a different point of view. Clin. Colorectal Cancer.

[9] Harry B.L., Smith M.L., Burton J.R. (2012). Medullary thyroid cancer and pseudocirrhosis: case report and literature review. Curr. Oncol..

[10] Adler M., Tang I., Gach M.W., MacFaul G. (2019). Recurrent metastatic breast cancer presenting with portal hypertension and pseudocirrhosis. BMJ Case Rep..

[11] Schreiner S.A., Gorman B., Stephens D.H. (1998). Chemotherapy-related hepatotoxicity causing imaging findings resembling cirrhosis. Mayo Clin. Proc..

[12] Agha R.A., Franchi T., Sohrabi C., Mathew G., for the SCARE Group (2020). The SCARE 2020 guideline: updating consensus surgical CAse REport (SCARE) guidelines. Int. J. Surg..

